# Identifying Candidates for Biopsy Omission in Prostate Cancer Using mpMRI and PSMA PET/CT

**DOI:** 10.1016/j.euros.2026.03.012

**Published:** 2026-07-13

**Authors:** Barış Esen, Yakup Kordan, Dilek Ertoy Baydar, Mehmet Onur Demirkol, Bengi Gürses, Metin Vural, Ahmet Furkan Sarıkaya, Umut Can Karaarslan, Mustafa Müdüroğlu, Serdar Madendere, Derya Tilki, Tarık Esen

**Affiliations:** a Department of Urology, Koc University Hospital, Istanbul, Türkiye; b Department of Urology, Emory University School of Medicine, Atlanta, USA; c Department of Pathology, Koc University Hospital, Istanbul, Türkiye; d Department of Nuclear Medicine, Koc University Hospital, Istanbul, Türkiye; e Department of Radiology, Koc University Hospital, Istanbul, Türkiye; f Radiology Clinic, American Hospital, Istanbul, Türkiye; g Urology Clinic, American Hospital, Istanbul, Türkiye; h Department of Urology, University Hospital Hamburg-Eppendorf, Hamburg, Germany; i Martini-Klinik Prostate Cancer Center, University Hospital Hamburg-Eppendorf, Hamburg, Germany

**Keywords:** Prostate cancer, PSMA, PET, Adverse pathology, Biopsy, mpMRI, Predicting factors

## Abstract

**Introduction:**

The superior diagnostic capabilities of novel imaging modalities such as multiparametric prostate magnetic resonance imaging MRI (mpMRI) and prostate-specific membrane-antigen positron emission tomography/computed tomography (PSMA PET/CT) might provide data for consideration of a prostate biopsy-omitting approach in a selected subgroup of patients with prostate cancer.

**Methods:**

Patients who underwent radical prostatectomy (RP) for prostate cancer between December 2015 and December 2024 were retrospectively evaluated, and only patients who underwent mpMRI and PSMA PET/CT were included in the study. After recording patient characteristics (age, prostate-specific antigen [PSA], PSA density [PSAD]), mpMRI characteristics (Prostate Imaging Reporting And Data System [PI-RADS] score, number of lesions, size of the index lesion), and PSMA PET characteristics (prostatic SUVmax ≥12 = PRIMARY 5), adverse pathology (≥pT3a or pN1 or GG≥3) rates at RP were investigated. Multivariable logistic regression analysis was performed to determine the independent predictors of adverse pathology.

**Results:**

The median age and serum PSA of 413 patients included in the study were 64.9 years (interquartile range [IQR]: 58.3–69.9) and 7.0 ng/dl (IQR: 4.9–9.4). The RP pathology revealed that 248 out of 413 patients (60%) had adverse features (58 out of 413 had GG4 or 5 disease). The multivariable analysis revealed that age, serum PSAD, PI-RADS-5 score on mpMRI, and prostatic SUVmax ≥12 (PRIMARY-5) on PSMA PET were independent predictors of adverse pathology. Based on these results, the combination of PSAD, MRI, and PSMA PET has consistently resulted in adverse pathology: PSAD >0.15 ng/ml/cc AND PI-RADS-5 AND PRIMARY 5.

**Conclusions:**

A combination of serum PSAD, PI-RADS score on mpMRI, and PRIMARY score on PSMA PET/CT has consistently resulted in adverse pathology, indicating consideration of a biopsy-omitting approach if further studies endorse these findings.

## Take Home Message

A combination of clinical parameters and imaging characteristics from superior diagnostic modalities (multiparametric prostate magnetic resonance imaging and prostate-specific membrane-antigen positron emission tomography/computed tomography) can accurately detect patients with adverse pathology. Therefore, further prospective studies are required to test the efficacy of radical prostatectomy without a prior biopsy using this combination.

ADVANCING PRACTICE **What does this study add?**

This study identifies independent clinical and imaging predictors of adverse pathology in patients with prostate cancer undergoing radical prostatectomy. By integrating PSA density (>0.15 ng/mL/cc), mpMRI findings (PIRADS 5), and PSMA PET/CT parameters (SUVmax ≥12), we define a subgroup of patients consistently associated with adverse pathological outcomes. These findings provide a data-driven framework for future prospective studies evaluating imaging-based diagnostic pathways and potential biopsy-sparing strategies in carefully selected patients.

**Clinical Relevance**

In contrast to many other tumour types, histological confirmation of malignancy remains a standard step in the diagnostic pathway for prostate cancer prior to treatment. This article contributes to a growing body of evidence suggesting that clinical parameters can predict the presence of clinically significant disease with high accuracy, raising the question of whether biopsy might be safely omitted in selected cases. However, any effort to avoid unnecessary biopsies must be carefully balanced against potential disadvantages, including the loss of detailed tumour information that is essential for optimal surgical or radiotherapeutic planning. Associate Editor: Roderick C.N. van den Bergh.

**Patient Summary**

In recent years, better imaging modalities have become available to predict patients with prostate cancer who had worse oncological features. In this study, we found several clinical and imaging parameters that can effectively predict adverse pathology, and defined a combination of clinical and imaging features that can be used in further studies investigating the efficacy of the biopsy-omitting strategy before radical prostatectomy.

## 1 Introduction

Currently, a prostate biopsy is mandatory for the diagnosis of prostate cancer (PCa) before proceeding with any further treatment [1]. Although prostate biopsy can be considered easy, effective, and safe [2], it is still an invasive procedure and is associated with side effects, such as infectious complications, rectal bleeding, hematuria-hematospermia, pain, lower urinary tract symptoms, urinary retention, and even mortality [3]. Besides, the number of new cases is projected to increase to 2.9 million cases by 2040, posing a substantial economic and workforce burden [4]. These considerations have led to the intriguing question of whether prostate biopsy can be omitted in selected patients with overt prostate cancer in the era of superior imaging modalities such as multiparametric prostate MRI (mpMRI) and prostate-specific membrane-antigen positron emission tomography/computed tomography (PSMA PET/CT).

There are a limited number of studies investigating the outcomes of radical prostatectomy (RP) without a prior prostate biopsy [5], [6], [7]. These studies included different mpMRI and PSMA PET criteria for selecting patients for RP without a prostate biopsy and evaluated clinically significant PCa (csPCa) rates as their endpoint. Although the RP outcomes are promising with very high csPCa rates (98.2–100%), caution is needed when evaluating these studies due to their limited sample size and the RP outcomes. For such an approach, it is of utmost importance to select the patients who would benefit from definitive treatment. Therefore, we need to set the bar high for such a biopsy-free approach to avoid the risk of unnecessary RP. Herein, we aimed to define the independent predictors of adverse pathology and clinical scenarios that almost consistently result in adverse pathology to be used in such a biopsy-free approach.

## 2 Patients and methods

**Fig. 1:**
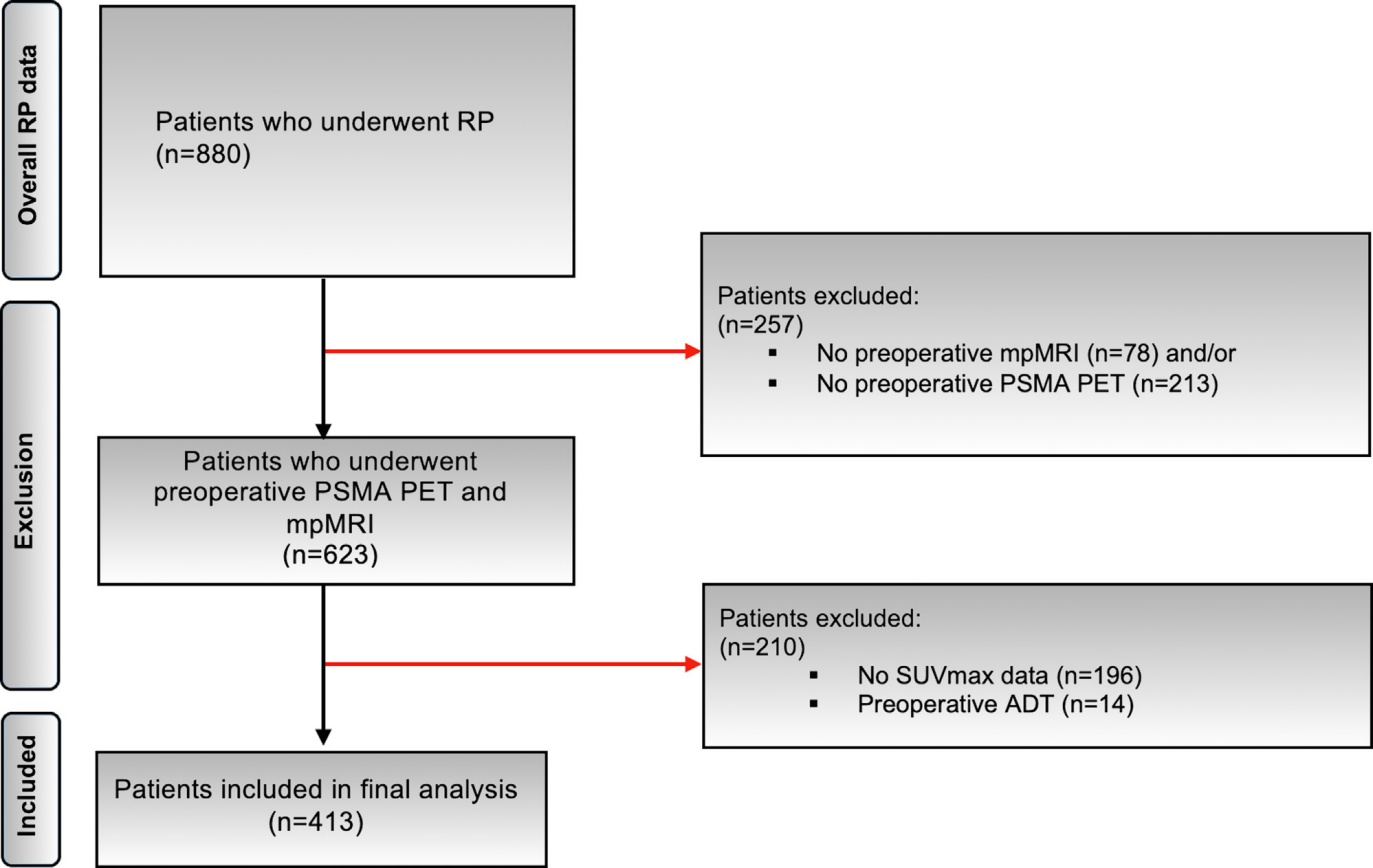
Flow diagram of patients included in the study.

Patients who underwent RP between December 2015 and December 2024 (*n* = 880) were retrospectively evaluated after the ethics committee approval. Patients who did not have a preoperative mpMRI (*n* = 78) and/or a PSMA PET for primary staging (*n* = 213) were excluded. Patients without any data on SUVmax at PSMA PET (*n* = 14) and those who received androgen deprivation therapy (ADT) before RP were also excluded. A total of 413 patients who met the inclusion criteria were further analyzed (Fig. 1). In all cases, mpMRI scans were performed before prostate biopsy, and PSMA PET scans were performed after the PCa diagnosis was made with prostate biopsy. Prostate biopsy was performed in all cases, but prostate biopsy-related parameters were not included in the analysis in line with the aims of this study. Details regarding the baseline characteristics of patients, including prostate biopsy data, are summarized in Supplementary Table 1. Patient characteristics such as age, body mass index (BMI), PSA values at diagnosis, and PSA density (PSAD) were recorded. All mpMRI and ^68^Ga-PSMA PET-related parameters, such as PI-RADS score and size of index lesion, multiplicity of lesions on mpMRI, and prostatic SUVmax on PSMA PET were evaluated. All surgeries were conducted by one of four highly-experienced urologic oncologic surgeons, each having performed over 500 robotic surgical cases, using either da Vinci Si or Xi robotic systems (Intuitive Surgical Incorporation, Sunnyvale, CA). Pathological examinations of RP specimens were performed by a team of three highly-experienced pathologists dedicated to uropathology. Adverse pathology was defined as International Society of Urological Pathologists (ISUP) grade ≥3 disease, and/or extraprostatic disease (≥pT3 or pN1 disease).

At our institution, mpMRI scans were performed using a 3-T device (Magnetom Skyra; Siemens AG, Munich) using a 16-channel pelvic phased array coil. External mpMRI scans were performed using either 1.5 or 3T devices. External mpMRI and PSMA PET scans were only accepted if centrally reviewed at our center or performed in a high-volume, experienced center. Scans that did not meet the required image and acquisition quality were excluded. All mpMRI scans were evaluated by uro-radiologists experienced in pMRI imaging, and PSMA PET scans were evaluated by highly-experienced nuclear medicine specialists. In all patients, prostatic lesions were categorized using the Prostate Imaging Reporting And Data System (PI-RADS) grades; PI-RADS 4–5 and PI-RADS ≤3 using either PI-RADS version 2.0 or version 2.1 [8]. The lesion with the highest PI-RADS score and largest size was identified as the index lesion on mpMRI. Prostate volume was measured using the ellipsoid formula (width × height × length × 0.52) on mpMRI. The PSMA PET/CT scans were performed using the tracer either ^68^Ga-PSMA-11 or ^68^Ga-PSMA-I&T at our center or other academic high-volume centers. All patients underwent ^68^Ga-PSMA PET for primary staging, and the highest SUVmax value within the prostate was documented. Patients were categorized into PRIMARY 1–4 (SUVmax <12), and PRIMARY 5 (SUVmax ≥12) [9].

Statistical analyses were conducted using SPSS software, version 24 (SPSS Inc., Chicago, IL, USA). Descriptive statistics were reported for all variables. Comparisons of means and medians were performed using either the Student’s *t* test or the Mann-Whitney U test, as appropriate. Proportions between groups were compared using the c^2^ test or Fisher’s exact test. A multivariable logistic regression analysis using the “enter” method on SPSS was performed to determine the independent predictors of adverse pathology. A bootstrap validation with 1000 resamples was performed to assess the internal stability of the multivariable model and correct for potential overfitting. Area under the curve (AUC) was calculated using receiver operating characteristic (ROC) curves for each predictor parameter and the “predicted possibilities” from logistic regression models. Results were presented with odds ratios (ORs) and 95% confidence intervals (CIs). PSAD was rescaled to per 0.1 unit increases to improve the interpretability of the ORs. A *p* value <0.05 was considered statistically significant.

## 3 Results

The median age and BMI of patients were 64.9 years (interquartile range [IQR]: 58.3–69.9) and 26.9 kg/m^2^ (IQR: 24.8–29.0). The median prostate size on mpMRI was 46 cc (IQR: 34–64). The median preoperative serum PSA and PSAD were 7.0 ng/dl (IQR: 4.9–9.4) and 0.15 (IQR: 0.09–0.22), respectively. The PI-RADS score of the index lesion was PI-RADS 1–2 in 13 (3.1%), PI-RADS 3 in 51 (12%), PI-RADS 4 in 215 (52%), and PI-RADS 5 in 134 (32%) patients. The median size of index lesions was 1.2 cm (IQR: 0.8–1.2). The median SUVmax on preoperative PSMA PET was 5.7 (IQR: 3.9–8.9). The SUVmax was ≥12 (PRIMARY-5) in 66 cases (16%). Final pathology of RP specimens revealed csPCa in 382 cases (92%); 237 had ISUP-2, 87 had ISUP-3, 22 had ISUP-4, and 36 had ISUP-5 disease. Overall, 208 patients had pT2 (50%), 157 had pT3 (38%), 45 had pT3b (11%), and three had pT4 (1%) disease. Pathological examination revealed adverse pathology in 248 patients (60%). Adverse pathology rates according to clinical and imaging characteristics are summarized in Table 1.

**Table 1:** Adverse pathology rates according to clinical and imaging characteristics

**Parameters**	**Adverse pathology, *n* (%)**
Age	
• <60 yr	57/119 (48%)
• 60–70 yr	124/188 (66%)
• ≥70 yr	67/106 (63%)
Serum PSA at diagnosis	
• ≤10 ng/dl	182/325 (56%)
• >10 ng/dl	66/88 (75%)
PSAD	
• ≤0.15	102/211 (48%)
• >0.15	146/202 (72%)
PIRADS score	
• PIRADS ≤3	29/64 (45%)
• PIRADS 4	112/215 (52%)
• PIRADS 5	107/134 (80%)
PRIMARY score	
• PRIMARY 1–4	193/348 (55%)
• PRIMARY 5	55/65 (85%)
**Clinical setting consistently results in adverse pathology**	
• PSAD > 0.15 ng/ml/m^2^ AND PIRADS-5 AND PRIMARY 5	22/22 (100%)

PIRADS = prostate imaging reporting and data system; PSA = prostate specific antigen; PSAD = prostate specific antigen density; REF = reference; OR = odds ratio.

**Fig. 2:**
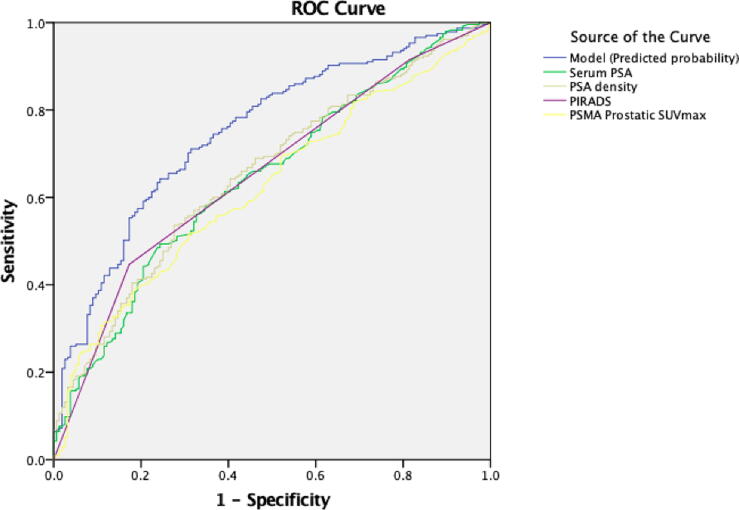
ROC analysis of serum PSA, PSAD, PI-RADS score on mpMRI, prostatic SUVmax on PSMA PET/CT, and the “adverse pathology predicted probabilities” of the logistic regression model.

Multivariable analysis revealed that age, serum PSAD, PI-RADS-5 score on mpMRI, and SUVmax ≥12 (PRIMARY-5) on PSMA PET were independent predictors of adverse pathology (Table 2). Receiver operating curves for predicting adverse pathology revealed AUCs of 0.644 for serum PSA, 0.654 for serum PSAD, 0.644 for PI-RADS score on mpMRI, and 0.619 for prostatic SUVmax on PSMA PET, respectively. The model achieved an AUC of 0.749 (95% CI: 0.701–0.798), which is higher than each of the singular parameters (Fig. 2). The bootstrap analysis results confirmed the robustness of the independent predictors, with minimal bias observed across all significant variables in the model (Supplementary Table 3).

**Table 2:** Multivariable logistic regression analysis to predict adverse pathology

**Parameters**	***p* value**	**OR**	**95% CI**	
			**Lower**	**Upper**
Age	0.016 a	1.05	1.01	1.08
PSA at diagnosis	0.4	0.97	0.93	1.06
PSAD^b^	0.005 a	1.56	1.14	2.12
mpMRI index lesion PIRADS score				
• PIRADS ≤3	REF			
• PIRADS 4	0.4	1.35	0.70	2.62
• PIRADS 5	0.001 a	3.66	1.73	7.11
mpMRI multiplicity (ref. solitary vs multiple)	0.15	0.71	0.45	1.13
PRIMARY score on PSMA PET (ref. PRIMARY 1–4 vs PRIMARY-5)	0.003 a	3.25	1.49	7.11

CI = confidence interval; mpMRI = multiparametric prostate magnetic resonance imaging; PIRADS = prostate imaging reporting and data system; PSA = prostate specific antigen; PSAD = prostate specific antigen density; REF = reference; OR = odds ratio.

a Statistically significant.

b PSAD was rescaled per 0.1 unit increases to improve the interpretability of the odds ratios.

**Fig. 3:**
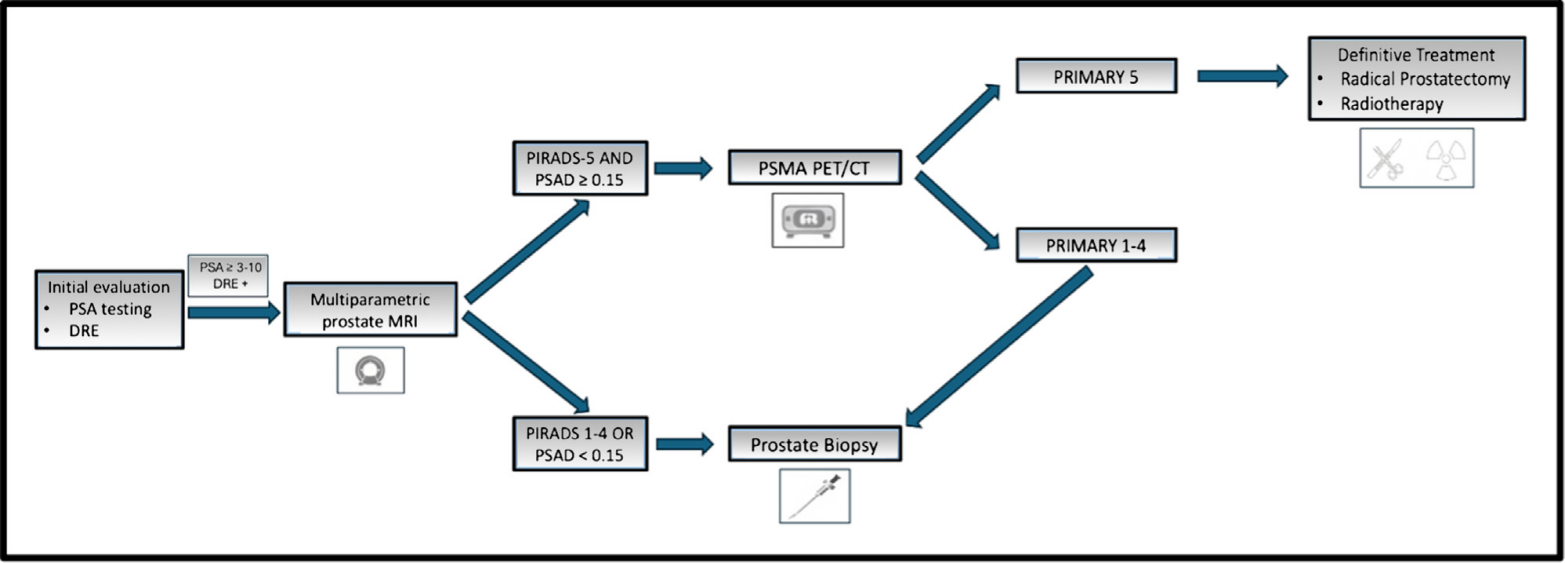
The proposed diagnostic workflow to omit prostate biopsy in the era of multiparametric prostate MRI and PSMA PET.

To facilitate clinical interpretation of the multivariable regression results, we further explored combinations of the independent predictors regarding adverse pathology rates. To maximize clinical utility, we focused on clinical parameters excluding age. Based on clinical independent predictors on the multivariable model, PSAD >0.15 ng/ml/m^2^ and PI-RADS-5 and PRIMARY 5 (SUVmax ≥12) represented a subgroup that consistently demonstrated adverse pathology (22 out of 22, 100%). Derived from these multivariable findings, Fig. 3 illustrates a potential diagnostic workflow using the independent predictors to facilitate clinical decision-making. Among patients who met these criteria, 12 (12/22, 55%) had GS 8–10 disease with pT3/4 disease, and six patients (27%) had pN1 disease. This specific combination yielded a specificity and positive predictive value of 100%, with a sensitivity of 9% and a negative predictive value of 42%. When prostate biopsy GG is included in the multivariable analysis, it was also found to be a dependent predictor of adverse pathology and to further improve the AUC (0.828, 95 CI: 0.788–0.868) of the logistic regression model (Supplementary Table 2 and Supplementary Fig. 1). The model including prostate biopsy GG but excluding mpMRI and PSMA PET-related parameters, had an AUC of 0.799 (95%CI: 0.757–0.841).

## 4 Discussion

The novel concept to perform RP without a biopsy is derived from several scientific data. First, the novel imaging modalities such as mpMRI and PSMA PET have significantly improved the detection of csPCa over conventional imaging with CT and bone scan [10], [11]. Although mpMRI and PSMA PET are not perfect for detecting csPCa, the detection rate increases significantly when used in combination [12]. Herein, we also show that PI-RADS-5 score on mpMRI and SUVmax ≥12 on PSMA PET are independent predictors of adverse pathology. However, the positive predictive value of the PI-RADS-5 score on mpMRI and the PRIMARY-5 score on PSMA PET were 80% and 85%, respectively, indicating a need for complementary use to detect adverse pathology along with previously known clinical predictors such as PSAD [13]. Secondly, the prostate biopsy is also not perfect in representing the nature of PCa, with significant discordance between prostate biopsy and RP specimens even in the era of targeted biopsy [14]. Besides, prostate biopsy is an invasive procedure associated with significant infectious and noninfectious complications despite lower reported infection rates with the transperineal route and by reducing the number of cores with novel targeted biopsy-only approaches (such as in-bore biopsy) without systematic biopsies [15], [16]. Since higher hemorrhagic complications requiring intervention were reported in patients who underwent prostate biopsy under anticoagulants, and patients with a high risk of stopping anticoagulants might represent a specific subgroup of patients who might benefit from a no-biopsy approach to avoid the need to discontinue anticoagulants twice, both before prostate biopsy and RP [17]. Finally, >2 million prostate biopsies are performed annually in the United States of America (USA) and Europe posing a significant economic and workforce burden [3]. As the incidence of PCa increases exponentially, omitting prostate biopsy in patients with overt PCa in the era of mpMRI and PSMA PET/CT may be one of the solutions to relieve this burden.

Nevertheless, possible drawbacks of such an approach should be noted. Initially, prostate biopsy provides clinically very important histological data (ISUP grade, presence of variant histology, presence of neurovascular invasion etc.) to optimize treatment (optimal patients for active surveillance or definitive treatment) in patients with PCa. High-risk GG4 or GG5 disease is highly unlikely to be downgraded to clinically insignificant disease after RP. Second, the relatively limited inter-reader reliability of the PI-RADS scoring system raises concerns about relying solely on imaging to forgo prostate biopsy in the diagnostic pathway. Biopsy functions as a safeguard against suboptimal or inaccurately interpreted mpMRI studies [18]. While these concerns are justified, the variability in mpMRI acquisition and reporting across centers is expected to narrow with the rapid advancement of artificial intelligence (AI)–based support tools [19]. Additionally, PSMA PET can serve as a complementary modality to corroborate mpMRI findings. Previous studies investigating RP without a prostate biopsy selected different mpMRI and PSMA PET criteria for inclusion and reported csPCa rates in RP specimens. Any disease ≥ISUP 2 disease was considered csPCa. Although the reported csPCa rates were very promising (98.2–100%), a significant percentage of patients had pT2 and/or ISUP 2 disease in these studies (Table 3). However, 29-year outcomes of the SPCG-4 study revealed that the risk of death from PCa among men with ISUP 2 is similar to that of those with a Gleason score of ISUP 1, and five times lower than patients with an ISUP 3 [20]. Current guidelines recommend AS in selected patients with ISUP 2 disease, and these patients underwent RP without the chance to consider AS with such an approach. Therefore, more stringent criteria should be adopted when selecting patients for RP without a prior biopsy to ensure that these patients require definitive treatment. From our large single-center experience, we found that patients with a PSAD ≥0.15 and a PI-RADS-5 lesion on mpMRI and a prostatic SUVmax ≥12 on PSMA PET have consistently resulted in adverse pathology and can be utilized as the inclusion criteria in further prospective studies. Beyond statistical significance, the clinical utility of our criteria is particularly demonstrated by its high specificity (100%) and positive predictive value (100%) when PSAD >0.15 ng/ml/m^2^, PI-RADS-5, and SUVmax ≥12 are combined. In practice, this “triple-positive” profile provides a reliable threshold for clinicians to identify patients at high risk who may benefit from definitive treatment even if prostate biopsy data is not available. Although such an approach may seem more appropriate for patients demanding surgery, since the need and duration of ADT in patients receiving radiation treatment are decided using prostate biopsy data. But a no-biopsy approach might also be adopted in patients who will receive radiotherapy if such an approach proves that imaging-based criteria reliably show adverse pathology in patients with PCa. Patients undergoing surgery should be well informed about the possible need for adjuvant or salvage radiotherapy after surgery. Although meta-analysis by the ARTISTIC collaboration found no oncological benefit of adjuvant radiotherapy over early salvage treatment [21], patients with high risk for recurrence, such as pN1 or GS 8–10 and pT3/4 disease, might still benefit from adjuvant radiotherapy [22]. Of patients who met our proposed adverse pathology criteria, 12 out of 22 had GS 8–10 disease, and six had pN1 disease, indicating high risk for recurrence. The criteria that we proposed to predict adverse pathology require both mpMRI and PSMA PET data without performing a prostate biopsy, raising an important question of how and to whom PSMA PET should be requested without a prostate biopsy. Since performing a PSMA PET on all biopsy-eligible patients would have caused an enormous financial toxicity, we propose a possible diagnostic workflow for such an approach (Fig. 3). It is important that only 8% of cases in our study met these criteria, underlining that this subgroup represents a minority of patients.

**Table 3:** Studies reporting outcomes of radical prostatectomy without a prostate biopsy

**First author (yr)**	**Design**	***N***	**Imaging criteria**	**RP outcome**
Meissner (2022) [5]	R	25	mpMRI: PIRADS ≥4 PSMA PET: PET score ≥4 and SUVmax≥4	25/25 (100%) csPca; • 15/25 pT2 • 6/25 pT3a • 4/25 pT3b • 8/25 ISUP 2 • 15/25 ISUP 3 • 2/25 ISUP ≥4
Niu (2024) [6]	P	47	mpMRI: PIRADS ≥4 PSMA PET: PET score ≥2	47/47 (100%) csPca; • 12/47 ISUP 2 • 19/47 ISUP 3 • 16/47 ISUP ≥4
Wang (2025) [7]	P	57	USTC prediction nomogram (mpMRI & PSAD) ≥0.60 PSMA PET: PET score ≥4 and SUVmax≥10	56/57 (98.2%) csPca; • 41/57 pT2 • 6/57 pT3a • 10/57 pT3b • 1/57 ISUP1 • 14/57 ISUP2 • 14/57 ISUP 3 • 28/57 ISUP ≥4

csPCa = clinically significant prostate cancer; mpMRI = multiparametric prostate magnetic resonance imaging; P = prospective; PIRADS = prostate imaging reporting and data system; PSAD = prostate specific antigen density; R = retrospective; RP = radical prostatectomy; SUV = standardized uptake value; USTC = University of Science and Technology of China.

This study has several limitations and strengths. The first and most important limitation is the retrospective nature of the study, which inherently relies on the completeness and accuracy of medical records, which may introduce selection bias due to the exclusion of patients with missing data, and unmeasured confounding factors that cannot be fully accounted for despite multivariable adjustment. Second, the study population was highly selected and relatively homogeneous, with a high prevalence of csPCa and adverse pathology. This may have influenced the discriminative performance of individual predictors and the observed AUC values. Third, as a single-center study including only patients with available PI-RADS and SUVmax data, the findings may not be fully generalizable to other populations or centers. The main strength is the homogenous patient population included in the study. All patients underwent mpMRI and PSMA PET before receiving any treatment. All pathological specimens were evaluated by experienced uropathologists, and only mpMRI and PSMA PET/CT scans performed in experienced centers were included in the study, limiting the problems regarding image quality and interpretation errors. However, since this is a single-center experience and included only patients with available PI-RADS assessment and prostatic SUVmax data, external validation of these findings is warranted to confirm the robustness and generalizability before being adopted as inclusion criteria of future prospective studies.

## 5 Conclusion

In conclusion, our study identified a very specific subgroup of patients consistently associated with adverse pathology at RP using a combination of PSAD, PI-RADS score on mpMRI, and SUVmax on PSMA PET. These outcomes are in line with previous studies demonstrating the diagnostic efficacy of novel imaging modalities in identifying csPCa, which may inform future investigations into biopsy-sparing strategies in carefully selected patients.

  ***Author contributions***: Barış Esen had full access to all the data in the study and takes responsibility for the integrity of the data and the accuracy of the data analysis.

*Study concept and design*: XX.

*Acquisition of data*: Surname, XX, XX.

*Analysis and interpretation of data*: Surname, XX, XX.

*Drafting of the manuscript*: Surname, XX, XX.

*Critical revision of the manuscript for important intellectual content*: Surname, XX, XX.

*Statistical analysis*: Surname, XX, XX or None.

*Obtaining funding*: Surname, XX, XX or None.

*Administrative, technical, or material support*: Surname, XX, XX or None.

*Supervision*: Surname, XX, XX or None.

*Other* (specify): [information from authorship form or None].

  ***Financial disclosures:*** Barış Esen certifies that all conflicts of interest, including specific financial interests and relationships and affiliations relevant to the subject matter or materials discussed in the manuscript (eg, employment/affiliation, grants or funding, consultancies, honoraria, stock ownership or options, expert testimony, royalties, or patents filed, received, or pending), are the following: None.

  ***Ethics statement:*** The study was conducted in accordance with the principles of the Helsinki Declaration. Institutional ethics committee approval was obtained.

  ***Disclosure statement*:** The authors declare that they have no known competing financial interests or personal relationships that could have appeared to influence the work reported in this paper.

  ***Data availability statement:*** The datasets generated during and/or analyzed during the current study are not publicly available due to ethical restrictions, but are available from the corresponding author on reasonable request.
