# Supplementary material for: Identifying Candidates for Biopsy Omission in Prostate Cancer Using mpMRI and PSMA PET/CT
Source: Eur Urol Open Sci. 2026 Jul 13;90:146–53. doi: 10.1016/j.euros.2026.03.012 (PMC13382317; doi:10.1016/j.euros.2026.03.012)

**Supplementary Table 1:** Demographics, clinical, pathological, and imaging characteristics of patients included in the study

| **Parameter** | **Median (IQR) / Frequency (%)** |
| --- | --- |
| **Age (years)** | 65 (59 – 70) |
| **BMI (kg/m^2^)** | 27 (25 – 29) |
| **Serum PSA (ng/mL)** | 7.0 (4.9 – 9.4) |
| **Serum PSAD (ng/mL)** | 0.15 (0.10 – 0.22) |
| **mpMRI PIRADS score** |  |
| - **PIRADS 1-2** | 13 (3.1%) |
| - **PIRADS 3** | 51 (12%) |
| - **PIRADS 4** | 215 (52%) |
| - **PIRADS 5** | 134 (32%) |
| **Size of index lesion (mm)** | 12 (8 – 17) |
| **Multiplicity of index lesions** | 141 (36%) |
| **Prostate biopsy technique** |  |
| - **Systematic biopsy** | 115 (28%) |
| - **MRI/US fusion or cognitive fusion biopsy** | 226 (55%) |
| - **In-bore biopsy** | 72 (17%) |
| **Total number of cores** |  |
| - **Systematic biopsy** | 12 (12 – 14) |
| - **MRI/US fusion or cognitive fusion biopsy** | 14 (13 – 15) |
| - **In-bore biopsy** | 4 (4 – 5) |
| **Prostate biopsy ISUP GG** |  |
| - **GG1** | 82/412 (20%) |
| - **GG2** | 171/412 (42%) |
| - **GG3** | 84/412 (20%) |
| - **GG4** | 52/412 (13%) |
| - **GG5** | 23/412 (5.6%) |
| **PSMA PET Prostatic SUVmax** | 5.7 (3.9 – 8.9) |
| **RP ISUP GG** |  |
| - **GG1** | 31/413 (7.5%) |
| - **GG2** | 237/413 (57%) |
| - **GG3** | 87/413 (21%) |
| - **GG4** | 22/413 (5.3%) |
| - **GG5** | 36/413 (8.7%) |
| **RP Pathological stage** |  |
| - **pT2** | 208/413 (50%) |
| - **pT3a** | 157/413 (38%) |
| - **pT3b** | 45 (11%) |
| - **pT4** | 3 (0.7%) |

**Abbreviations:** BMI: body mass index, **CI:** confidence interval, IQR: interquartile range, **mpMRI:** multiparametric prostate magnetic resonance imaging, GG: grade group, ISUP: International Society of Urological Pathology, **PIRADS:** prostate imaging reporting and data system, PSA: prostate specific antigen, **PSAD:** prostate specific antigen density, REF: reference, RP: radical prostatectomy, SUV: standardized uptake value, US: Ultrasound

**Supplementary Table 2:** Multivariable logistic regression analysis to predict adverse pathology including prostate biopsy data

| **Parameters** | **p-value** | **OR** | **95% CI** | |
| --- | --- | --- | --- | --- |
|  |  |  | **Lower** | **Upper** |
| Age | 0.016* | 1.05 | 1.01 | 1.08 |
| PSA at diagnosis | 0.4 | 0.97 | 0.90 | 1.04 |
| PSAD^Ψ^ | 0.010* | 1.54 | 1.11 | 2.14 |
| mpMRI index lesion PIRADS score |  |  |  |  |
| - PIRADS ≤3 | REF |  |  |  |
| - PIRADS 4 | 0.9 | 1.07 | 0.51 | 2.24 |
| - PIRADS 5 | 0.007* | 3.10 | 1.35 | 7.11 |
| mpMRI multiplicity (ref. solitary vs multiple) | 0.19 | 0.71 | 0.43 | 1.18 |
| PRIMARY score on PSMA PET (ref. PRIMARY 1-4 vs PRIMARY-5) | 0.034* | 2.52 | 1.07 | 5.93 |
| Prostate biopsy GG |  |  |  |  |
| - GG1 | REF |  |  |  |
| - GG2 | 0.033 | 1.98 | 1.06 | 3.70 |
| - GG3 | <0.001* | 4.84 | 2.27 | 10.32 |
| - GG4 or 5 | <0.001* | 54.7 | 12.0 | 250 |

* Statistically significant

Ψ PSAD was rescaled per 0.1 unit increase to improve the interpretability of the odds ratios

**Abbreviations:** **CI:** confidence interval, **mpMRI:** multiparametric prostate magnetic resonance imaging, GG: grade group, **PIRADS:** prostate imaging reporting and data system, PSA: prostate specific antigen, **PSAD:** prostate specific antigen density, REF: reference, **OR: odds ratio**

**Supplementary Table 3:** Bootstrap analysis of the multivariable analysis showing very low bias, and predictors remained robust after 1000 iterations

| **Variable** | **B** | **Bias** | **Std. Error** | **Sig. (2-tailed)** | **BCa 95% Confidence Interval (Lower)** | **BCa 95% Confidence Interval (Upper)** |
| --- | --- | --- | --- | --- | --- | --- |
| Age | .044 | .001 | .018 | .010 | .01 | .08 |
| PSA ng/dL | -.009 | .006 | 0.040 | 0.813 | -.07 | .10 |
| PSAD | .442 | .008 | .179 | .010 | .10 | 0.80 |
| PIRADS-4 (vs PIRADS-≤3) | .300 | -.002 | .333 | .357 | -.37 | .96 |
| PIRADS-5 (vs PIRADS-≤3) | 1.298 | .016 | .408 | .002 | .52 | 2.08 |
| mpMRI multiplicity (ref. solitary vs multiple) | -0.342 | -0.012 | 0.431 | 0.004 | 0.47 | 2.17 |
| SUVmax (ref SUVmax<12 vs ≥12) | 1.179 | .050 | .431 | .004 | .469 | 2.171 |
| **Constant** | 1.926 | .022 | 1.056 | .070 | -4.292 | .205 |

**Abbreviations:** BCa: bias-corrected and accelerated, **mpMRI:** multiparametric prostate magnetic resonance imaging, GG: grade group, **PIRADS:** prostate imaging reporting and data system, PSA: prostate specific antigen, **PSAD:** prostate specific antigen density, REF: reference, Sig.: significance, SUVmax: maxinum standardized uptake value, **OR: odds ratio**

**Supplementary Table 4:** Multivariate logistic regression analysis to predict adverse pathology using only clinical parameters and prostate biopsy data

| **Parameters** | **p-value** | **OR** | **95% CI** | |
| --- | --- | --- | --- | --- |
|  |  |  | **Lower** | **Upper** |
| Age | 0.002* | 1.05 | 1.02 | 1.09 |
| PSA at diagnosis | 0.5 | 0.98 | 0.91 | 1.04 |
| PSAD | 0.001* | 1.74 | 1.27 | 2.39 |
| Prostate biopsy GG |  |  |  |  |
| - GG1 | REF |  |  |  |
| - GG2 | 0.025* | 1.93 | 1.08 | 3.44 |
| - GG3 | <0.001* | 4.56 | 2.27 | 9.13 |
| - GG4 or 5 | <0.001* | 41.9 | 11.8 | 148 |

**Abbreviations:** **CI:** confidence interval, **mpMRI:** multiparametric prostate magnetic resonance imaging, GG: grade group, PSA: prostate specific antigen, **PSAD:** prostate specific antigen density, REF: reference, **OR: odds ratio**

**Supplementary Figure 1:** ROC analysis of “adverse pathology predicted probabilities” of the logistic regression model, including prostate biopsy ISUP grade group (Area under curve 0.828, 95%CI: 0.788 – 0.868)


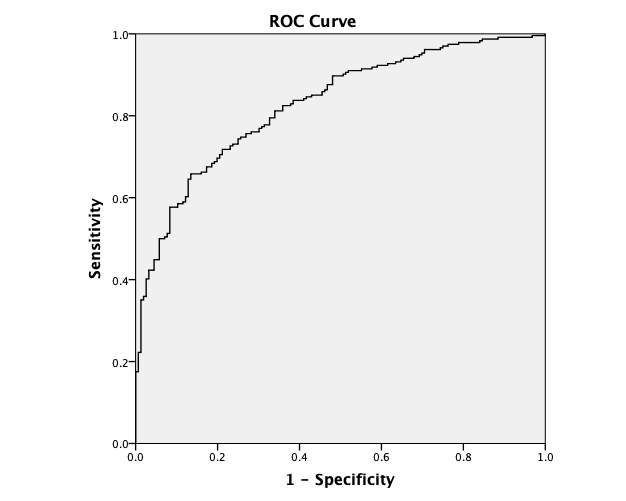


**Supplementary Figure 2:** ROC analysis of “adverse pathology predicted probabilities” of the logistic regression model, including prostate biopsy ISUP grade group but excluding imaging parameters (Area under curve 0.799, 95%CI: 0.757 – 0.841)


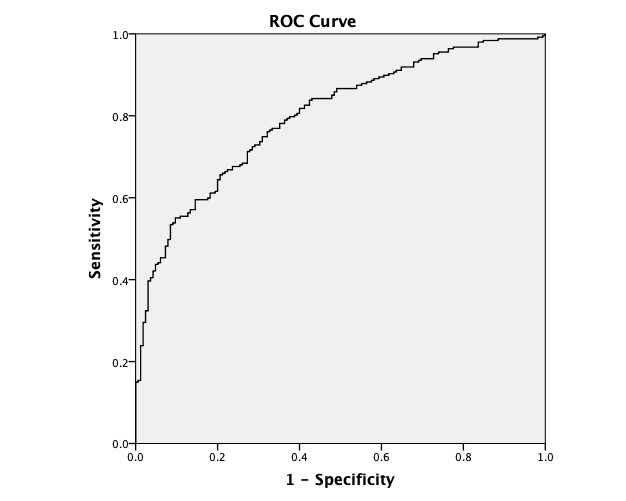

Supplement: Supplementary Data 1 [file mmc1.docx]
